# Isolation of goat milk small extracellular vesicles by novel combined bio-physical methodology

**DOI:** 10.3389/fbioe.2023.1197780

**Published:** 2023-09-27

**Authors:** María Isabel González, Begoña Gallardo, Carlos Cerón, Elena Aguilera-Jiménez, Marta Cortes-Canteli, Héctor Peinado, Manuel Desco, Beatriz Salinas

**Affiliations:** ^1^ Unidad de Medicina y Cirugía Experimental, Instituto de Investigación Sanitaria Gregorio Marañón, IiSGM, Madrid, Spain; ^2^ Unidad de Imagen Avanzada, Centro Nacional de Investigaciones Cardiovasculares (CNIC) Carlos III, Madrid, Spain; ^3^ Cardiovascular Risk Factors and Brain Function Programme, Centro Nacional de Investigaciones Cardiovasculares (CNIC) Carlos III, Madrid, Spain; ^4^ Instituto de Investigación Sanitaria Fundación Jiménez Díaz (IIS-FJD), Madrid, Spain; ^5^ Laboratorio de Microambiente y Metástasis, Departamento de Oncología Molecular, Centro Nacional de Investigaciones Oncológicas (CNIO) Carlos III, Madrid, Spain; ^6^ Departamento de Bioingeniería, Universidad Carlos III de Madrid, Madrid, Spain; ^7^ CIBER de Salud Mental, Instituto de Salud Carlos III, Madrid, Spain

**Keywords:** goat milk, exosomes, small extracellular vesicles, isolation methods, differential ultracentrifugation, exosome characterization

## Abstract

**Introduction:** Goat milk is notable as a cost-effective source of exosomes, also known as small extracellular vesicles (sEVs). These nanoparticle-like structures are naturally secreted by cells and have emerged as potential diagnostic agents and drug delivery systems, also supported by their proven therapeutic effects. However, the complexity of goat milk and the lack of standardized protocols make it difficult to isolate pure sEVs. This work presents an optimized approach that combines well-established physical isolation methods with the biological treatment of milk with rennet.

**Methods:** sEVs derived from goat milk were purified using a methodology that combines differential ultracentrifugation, rennet, and size-exclusion chromatography. This novel strategy was compared with two of the main methodologies developed for isolating extracellular vesicles from bovine and human milk by means of physico-chemical characterization of collected vesicles using Transmission Electron Microscopy, Western blot, Bradford Coomassie assay, Dynamic Light Scattering, Nanoparticle Tracking Analysis and Zeta Potential.

**Results:** Vesicles isolated with the optimized protocol had sEV-like characteristics and high homogeneity, while samples obtained with the previous methods were highly aggregated, with significant residual protein content.

**Discussion:** This work provides a novel biophysical methodology for isolating highly enriched goat milk sEVs samples with high stability and homogeneity, for their further evaluation in biomedical applications as diagnostic tools or drug delivery systems.

## 1 Introduction

Extracellular vesicles (EVs) are lipid bilayer-based particles that are naturally secreted by cells and serve as mediators in cell-to-cell communication. Different vesicle types can be distinguished according to their cellular origin, morphology, size, and biological function [1]. From a structural point of view, the term small extracellular vesicle (sEV), also known as “exosome”, can be applied to bilayer membrane vesicles of nanometric size [100–200 nm, according to Minimal Information for Studies of Extracellular Vesicles (MISEV) 2018 recommendations (Théry et al., 2018)] and cup-shape morphology (Abels and Breakefield, 2016; de la Torre Gomez et al., 2018). These nanovesicles have an endosomal origin and their cargo is composed of lipids, metabolites, and nucleic acids (mainly miRNAs) (Sanwlani et al., 2020). Characteristic biomarkers of these small vesicles include tetraspanins (CD63, CD81) and other membrane molecules (e.g., TSG101) (Zhang et al., 2020). Several publications have suggested using sEVs in biomedical applications because they could overcome some limitations associated with widely used synthetic liposomes. Similar to these artificial formulations, the lipid bilayer of sEVs allows them to be loaded with both hydrophilic and hydrophobic drugs, supporting their use as drug delivery systems (DDS) (van der Meel et al., 2014). Additionally, they exhibit a longer circulation time in blood than liposomes and have an intrinsic ability to target specific cells and tissues (Tschuschke et al., 2020).

Despite all these promising properties, not all sEVs are available for biomedical purposes. The cellular origin determines not only the composition of the nanovesicle but also its biological function. Foodstuffs and plants have been purposed as attractive sources of sEVs regarding accessibility, cost-effectiveness, biocompatibility, and cross-species tolerance (Chen et al., 2021). Specifically, milk represents the only biological fluid commercially available and widely consumed by humans.

sEVs contained in milk are of particular interest because of their remarkable robustness under degradation conditions, which is necessary for them to be incorporated into the organism through the intestine after oral ingestion (Sanwlani et al., 2020). In addition, their inherent biological functionality supports a promising role as targeted agents; milk sEVs are involved in the regulation of inflammatory processes and immune responses as they provide a vehicle for miRNA transmission from mothers to infants (de la Torre Gomez et al., 2018). Several publications have reported efficient encapsulation of therapeutic agents, including antitumor drugs, in these vesicles (Munagala et al., 2016). Furthermore, the successful labeling of these nanovesicles with radioisotopes and fluorescent probes also supports their potential use as novel diagnostic agents for nuclear or near-infrared fluorescence imaging (González et al., 2020; Adriano et al., 2021; Santos-Coquillat et al., 2022).

Among the different mammalian milk sources, goat milk is notable for the significant effects of its sEVs on inflammatory pathways (Mecocci et al., 2020; Santos-Coquillat et al., 2022). Although goat milk is one of the milks most consumed by humans, goat milk vesicles have not been widely investigated, especially in comparison with bovine milk sEVs (Golan-Gerstl et al., 2017). One drawback of the use of goat milk sEVs for biomedical purposes is the high fat content of goat milk compared with those of other milks consumed by humans, as well as the significant casein content (Izumi et al., 2013), which could limit the efficiency of current protocols designed to isolate sEVs.

The purity and homogeneity of isolated sEVs are crucial, due to their direct influence on the biophysical features of these nanovesicles. For example, co-isolated residual proteins can bind to sEVs and change their surface charge, modifying their *in vivo* stability and natural tropism (Beit-Yannai et al., 2018; Midekessa et al., 2020). Various approaches are currently employed in the isolation of milk sEVs, including sequential centrifugation, size-exclusion chromatography, density gradient centrifugation, and immunomagnetic-bead precipitation (Yu et al., 2018; Sanwlani et al., 2020; Sedykh et al., 2020). Among them, differential ultracentrifugation (dUC) is one of the most widely used techniques for EV isolation (Théry et al., 2018). This physical separation technique allows the treatment of large milk volumes, increasing the amount of sEVs that can be recovered. Nevertheless, the co-precipitation of “contaminants” with nanovesicle-like physicochemical properties, such as proteins, casein, or fat-containing globules, limits the purity of the vesicles isolated with this method (Théry et al., 2018).

Size-exclusion chromatography (SEC) is an effective complementary method to remove part of those co-isolated milk components and improve the purity of milk sEVs (Wei et al., 2020). However, the combination of dUC with SEC is still not enough to eliminate milk components similar in size or morphology to the sEVs, such as casein and small lipoproteins, supporting the need to complement these methods with other, more selective techniques.

In this work we propose an optimized protocol for the isolation of goat milk sEVs that combines dUC with the innovative biological treatment of milk with microbial rennet to enhance the removal of casein and other milk proteins. Additionally, our protocol is complemented by SEC to improve the purity of the isolated goat milk sEVs, minimize their aggregation, and collect an enriched nanovesicle population as homogeneous as possible. To demonstrate the success of our technique in isolating highly enriched sEVs samples, we compared samples obtained through this methodology with samples collected using two previously published and well-known protocols for the isolation of EVs from bovine and human milk (Izumi et al., 2013; Gao et al., 2019), by means of a complete physicochemical characterization by Bradford Coomassie assay, Western blot, Transmission Electron Microscopy (TEM), Dynamic Light Scattering (DLS), Zeta Potential and Nanoparticles Tracking Analysis (NTA).

## 2 Materials and methods

Commercial, organic, semi-skim goat milk (El Cantero de Letur, Albacete, Spain) was acquired from the supermarket and stored at 4°C until use. All centrifugation steps were performed at 4°C, employing an AVANTI J-30I centrifuge, a Ja 30,50 Ti fixed-angle rotor (k factor = 280) and 30-mL polycarbonate tubes. This equipment was purchased from Beck-man Coulter Instruments (Brea, CA, United States). The starting volume of goat milk was 60 mL for each isolation procedure.

Unless otherwise noted, all reagents were purchased from Merck Life Science (Darmstadt, Germany) and used without further purification. Polyethersulfone (PES) membrane filters were acquired from Labbox Labware S.L. (Barcelona, Spain).

### 2.1 Isolation of small extracellular vesicles from goat milk

This section describes an optimized methodology for the efficient isolation of sEVs from commercial goat milk that combines physical and biological approaches. To assess the advantages of this procedure, goat milk was also treated with two other physical isolation protocols proposed by Izumi et al. and Gao et al. (Izumi et al., 2013; Gao et al., 2019) and previously employed in the isolation of EVs from bovine and human milk. The comparison was carried out through the complete physicochemical characterization of all the collected samples.

#### 2.1.1 Optimized protocol (biophysical procedure)

A 60-mL sample of commercial goat milk was divided into two 30-mL centrifuge tubes and centrifuged for 10 min at 5,000 × g to remove the milk fat and fat-containing vesicles that are commonly present. Defatted supernatants were tempered and treated for 20 min at 37°C with 150 µL of microbial rennet per centrifuged tube (Postres Ultzama, Na-varra, Spain). Milk samples were centrifuged for 10 min at 5,000 × g after the coagulation step. The resultant supernatants were successively centrifuged, first for 35 min at 13,000 × g and then for 15 min at 35,000 × g, to ensure the removal of mammary gland–derived cell debris, somatic cells, and large extracellular vesicles (i.e., microvesicles). Milk sEVs were precipitated by ultracentrifugation at 100,000 × g for 65 min. The whitish pellets were washed twice with 5 mL of phosphate-buffered saline (1X PBS) and ultracentrifuged at 100,000 × g for 95 min. The resultant pellets were pooled, brought to 2.5 mL volume with 1X PBS, and purified by SEC using PD-10 columns (GE Healthcare Bio-Sciences AB, Chicago, IL, United States). Briefly, sEVs were collected in 7 fractions of 500 µL of 1X PBS and analyzed by TEM. sEV-enriched fractions were mixed, brought to 5 mL volume with 1X PBS, and ultracentrifuged at 100,000 × g for 95 min. The resultant pellet of milk sEVs was dispersed in 100–200 µL of 1X PBS.

#### 2.1.2 Physical isolation by triple differential centrifugation and filtration (the physical 3-dUC procedure)

Based on the protocol described from Gao et al. (Gao et al., 2019), a 60-mL sample of commercial goat milk was divided into two 30-mL centrifuge tubes and successively centrifuged for 10 min at 2000 × g and 40 min at 12,000 × g to remove the fat layer and cell debris. Defatted supernatants were passed through a 0.22-µm PES membrane filter by vacuum filtration, and sEVs were precipitated by ultracentrifugation at 100,000 × g for 120 min. The sEV pellets were resuspended in 500 µL of 1X PBS.

#### 2.1.3 Physical isolation by six-fold differential ultracentrifugation and filtration (the physical 6-dUC procedure)

Based on the protocol described from Izumi et al. (Izumi et al., 2013), a 60-mL sample of commercial goat milk was divided into two 30-mL centrifuge tubes and centrifuged for 10 min at 1,200 × g to remove fat-containing vesicles, cells and large debris. Defatted supernatants were centrifuged twice at 21,500 × g for 30 min to eliminate casein and residual fat. Next, the resultant supernatants were centrifuged again at 21,500 × g for 60 min in order to remove the remaining casein. The milk whey was filtered through 0.65-, 0.45-, and 0.22-µm PES syringe membrane filters to step-wise eliminate residual contaminants. sEVs were precipitated by ultracentrifugation at 100,000 × g for 90 min. Pellets were washed with 5 mL of 1X PBS and ultracentrifuged at 100,000 × g for 90 min. Precipitated sEVs were dispersed in 200 µL of 1X PBS.

### 2.2 Physicochemical characterization of goat milk small extracellular vesicles

The products obtained from the three isolation procedures were fully characterized by physicochemical assays, following the MISEV 2018 recommendations (Théry et al., 2018). We employed triplicates of the samples acquired by each isolation methodology.

#### 2.2.1 Protein content determination

Total protein was quantified by Bradford-Coomassie colorimetric assay. Aliquots of 5 µL from each sEV sample, previously diluted 5-fold in 1X PBS, were loaded in a flat-bottom 96-well plate (ThermoFisher Scientific, Unitd States) and quantified against a bovine serum albumin standard curve (125–1,000 μg/mL). All samples were incubated for 10 min with 200 µL of ready-to-use Coomassie staining reagent. Absorbance per sample was measured at 540 nm using a 680 XR Microplate Reader (Bio-Rad Laboratories, Hercules, CA, United States).

#### 2.2.2 Transmission Electron Microscopy

The shape, size and homogeneity of isolated sEVs were assessed by TEM at the ICTS Centro Nacional de Microscopía Electrónica (Universidad Complutense de Madrid, Madrid, Spain). This technique also allowed the detection of impurities and aggregates. Aliquots of 30 µg of sEVs were placed over formvar carbon-coated copper grids and negatively stained at room temperature with uranyl acetate. Samples were imaged at different magnifications using a JEOL JEM-1010 microscope operating at 100 kV.

#### 2.2.3 Dynamic Light Scattering

DLS was selected for the analysis of the hydrodynamic size distribution of milk sEVs. Aliquots of 5 µL were diluted 200-fold in 1X PBS and placed in DTS0012 disposable cuvettes for sEV measurement in a Zetasizer Nano ZS90 (Malvern Panalytical, Malvern, United Kingdom). Three replicates per sample were recorded under the following parameters: 25°C, 9 runs, and 10 s/run. Hydrodynamic size was evaluated from intensity distributions.

#### 2.2.4 Zeta potential

The superficial charge of sEVs was evaluated by measuring the zeta potential (Z-potential) in a Zetasizer Nano ZS90 (Malvern Panalytical, Malvern, United Kingdom). The same samples as used for DLS were placed in DTS1070 cuvettes, and measurements were run at 25°C, recording three replicates per sample.

#### 2.2.5 Nanoparticle tracking analysis

The real-time concentrations (particle/mL) as well as core sizes of sEVs were determined using a NanoSight NS300 instrument (Malvern Panalytical, Malvern, United Kingdom), equipped with a high-sensitivity metal-oxide semiconductor (sCMOS) camera, a 532-nm laser, and NTA 3.4 Build software. Aliquots of 5 µL of milk sEVs were diluted 200-fold with 1X PBS and filtered through 0.45-µm PES membrane filters for technical requirement. Samples were then injected into the NanoSight chamber by use of a syringe pump module, establishing an infusion speed of 40. Three dynamic videos of 60 s were recorded per sample, setting the camera level to 12, the detection threshold to 5, and temperature to 25°C. Replicated histograms were averaged to determine the modal size and particle concentration.

### 2.3 Western blot analysis

The detection of the sEV protein markers TSG101 and CD81 in the samples obtained by the three isolation procedures was performed by Western blot assay. sEV aliquots were homogenized 1:1 in RIPA buffer (PBS with 1% Nonidet P-40, 0.5% sodium deoxycholate, 0.1% sodium dodecyl sulfate), and protease inhibitor cocktail (Roche, Basel, Switzerland). The homogenates were centrifuged at 10,000 × g and 4°C for 10 min, and the supernatants were transferred to different tubes. The amount of soluble protein was quantified with a Bicinchoninic Acid Kit for protein determination, following the manufacturer´s instructions (ThermoFisher Scientific, Rockford, IL, United States). Proteins were resuspended in reducing sodium dodecyl sulfate loading buffer and heated at 95°C for 5 min. Next, 15 µg of sEV proteins were run on a 10% polyacrylamide gel under reducing conditions and transferred to a polyvinylidene fluoride membrane (Immobilon-P, Merck, Darmstadt, Germany). Circulating sEVs isolated from human WM64 melanoma cells as previously described (Peinado et al., 2012) were run in parallel as a positive control. Membranes were blocked with 3% bovine serum albumin, then incubated with a rabbit polyclonal anti-TSG101 antibody or a mouse monoclonal anti-CD81 antibody (ThermoFisher Scientific, Waltham, MA, United States), both at a dilution of 1:1000 in blocking buffer, and finally incubated with the corresponding horseradish peroxidase–conjugated secondary antibodies (Agilent Dako, Santa Clara, CA, United States). Direct digital images were acquired with an ImageQuant LAS 4000 mini (GE Healthcare, Chicago, IL, United States).

### 2.4 Data analysis and statistical methods

Data processing, graphical representations and statistical analysis were carried out using Prism 9.0.0 Software (GraphPad Software, La Jolla, CA, United States). Data have been expressed as the mean ± SD.

Statistical analysis of Nanoparticle Tracking Analysis and Z-Potential data (both in Figure 7) were performed by one-way ANOVA and Tukey’s multiple comparison tests. In case of size comparisons by Dynamic Light Scattering and Nanoparticle Tracking Analysis, statistical analysis was carried out by two-way ANOVA and Tukey’s multiple comparison tests. All statistical tests were run after checking for normality. Differences were considered statistically significant for *p* values below 0.05. Significant differences stand for: * (*p* ≤ 0.05), ** (*p* ≤ 0.01), *** (*p* ≤ 0.001), **** (*p* ≤ 0.0001).

## 3 Results

### 3.1 Qualitative comparison of sEV isolation methodologies

**FIGURE 1:**
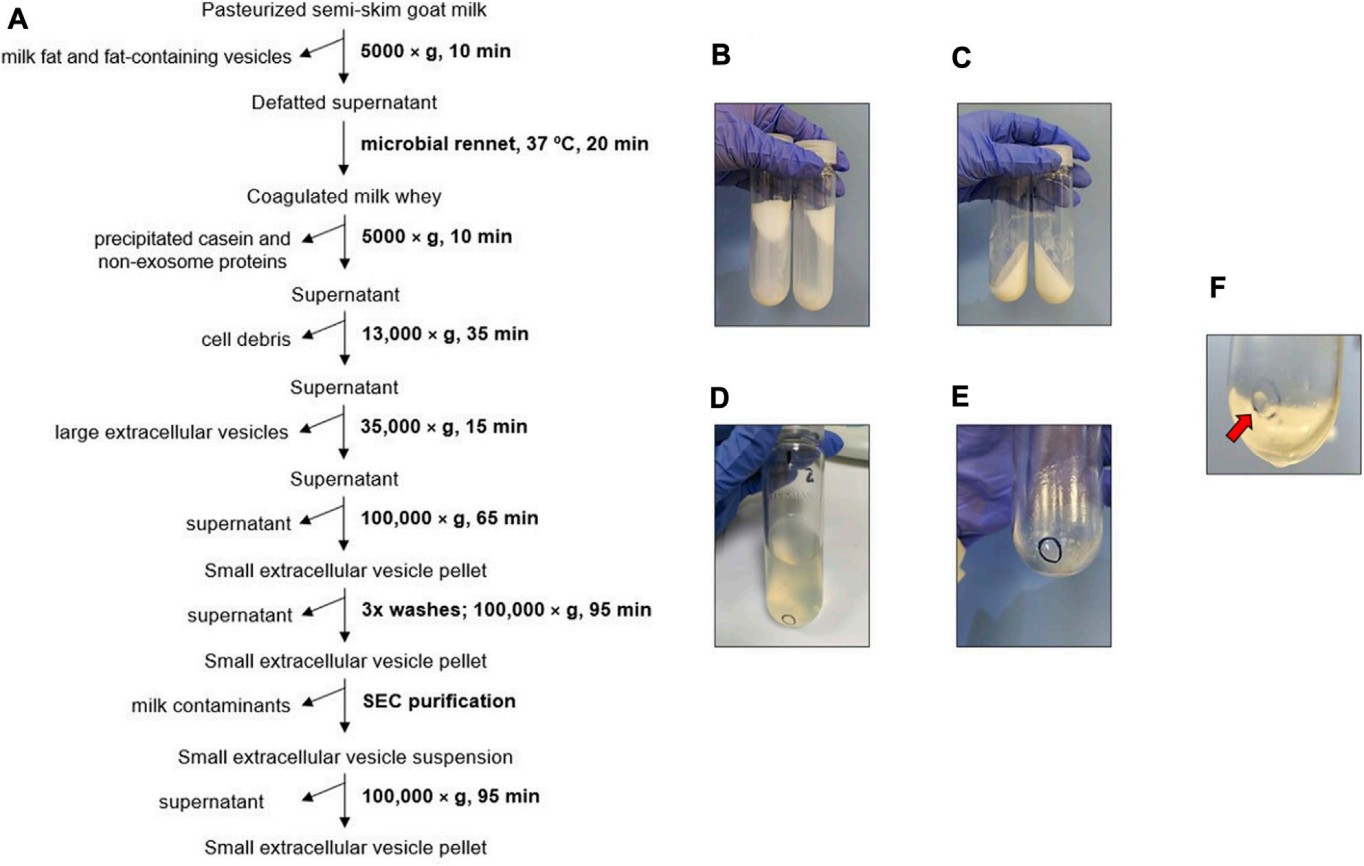
Biophysical procedure. **(A)** Scheme of goat milk small extracellular vesicle (sEV) isolation protocol. **(B)** Milk fat stuck to the walls of the centrifuge tube after the first centrifugation step. **(C)** Casein coagulated by microbial rennet. **(D)** Appearance of supernatants prior to sEV precipitation. **(E)** Pellet of cell debris and large extracellular vesicles. **(F)** Pellet of milk sEVs (red arrow).

The biophysical procedure (Figure 1A) combines the removal of fat milk, cell debris and large vesicles through the physical method of dUC with the biological precipitation of casein and residual proteins by the enzymatic activity of microbial rennet.

Milk fat and fat-containing vesicles were mainly separated from the milk whey in the first, low-speed centrifugation step (Figure 1B). The subsequent coagulation of the milk by microbial rennet precipitated a large amount of casein along with other residual proteins (Figure 1C). The resultant supernatants were transparent (Figure 1D). Cell debris and large EVs were collected after centrifuging at higher speeds (Figure 1E), and milk sEVs were finally precipitated at 100,000 × g. The washing steps allowed the elimination of co-isolated milk residues, clarifying the sEV pellet, which presented a whitish aspect.

**FIGURE 2:**
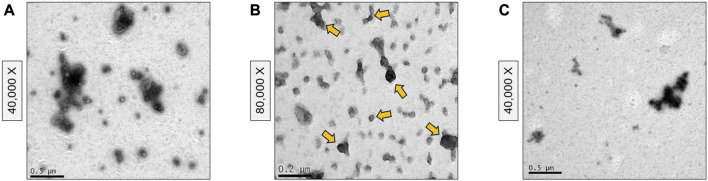
Transmission Electron Microscopy images of size-exclusion chromatography fractions. **(A)** First elution fraction containing large extracellular vesicles. **(B)** Intermediate elution fractions containing small extracellular vesicle-type vesicles (some of them marked with yellow arrows). **(C)** Last elution fraction containing residual proteins.

To assess the effect of including SEC in the goat milk sEV isolation protocol, eluted fractions were analyzed by TEM (Figure 2). Large extracellular vesicles were detected in the first elution fraction (500 µL), without the presence of sEV-like particles (Figure 2A). Intermediate elution fractions (2.5 mL) were clearly enriched in sEV-like nanovesicles (Figure 2B), and the last elution fraction (500 µL) mainly presented residual proteins (Figure 2C). Once the first and last fractions were discarded, milk sEVs were precipitated again, forming a gelatinous and transparent pellet (Figure 1F).

**FIGURE 3:**
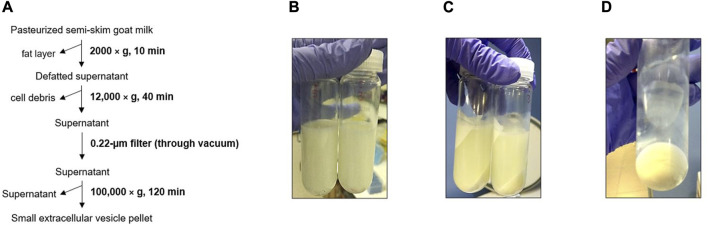
Triple differential centrifugation and filtration (physical 3-dUC) procedure. **(A)** Scheme of goat milk small extracellular vesicle (sEV) isolation protocol. **(B)** Defatted supernatant after first centrifuge step. **(C)** Precipitated milk contaminants. **(D)** Appearance of pelleted milk sEVs.

In the case of the physical 3-dUC procedure (Figure 3A), a lower amount of milk fat was observed stuck to the walls of the centrifuge tube in the first centrifugation step in comparison with the biophysical procedure (Figure 3B). This milk component continued to precipitate in the following isolation step, which resulted in cloudy supernatants (Figure 3C), and the density of these fluids hindered their filtration with vacuum. After the sEVs were precipitated, the resulting pellets appeared white and dense, and these precipitates were difficult to disperse with a pipette (Figure 3D).

**FIGURE 4:**
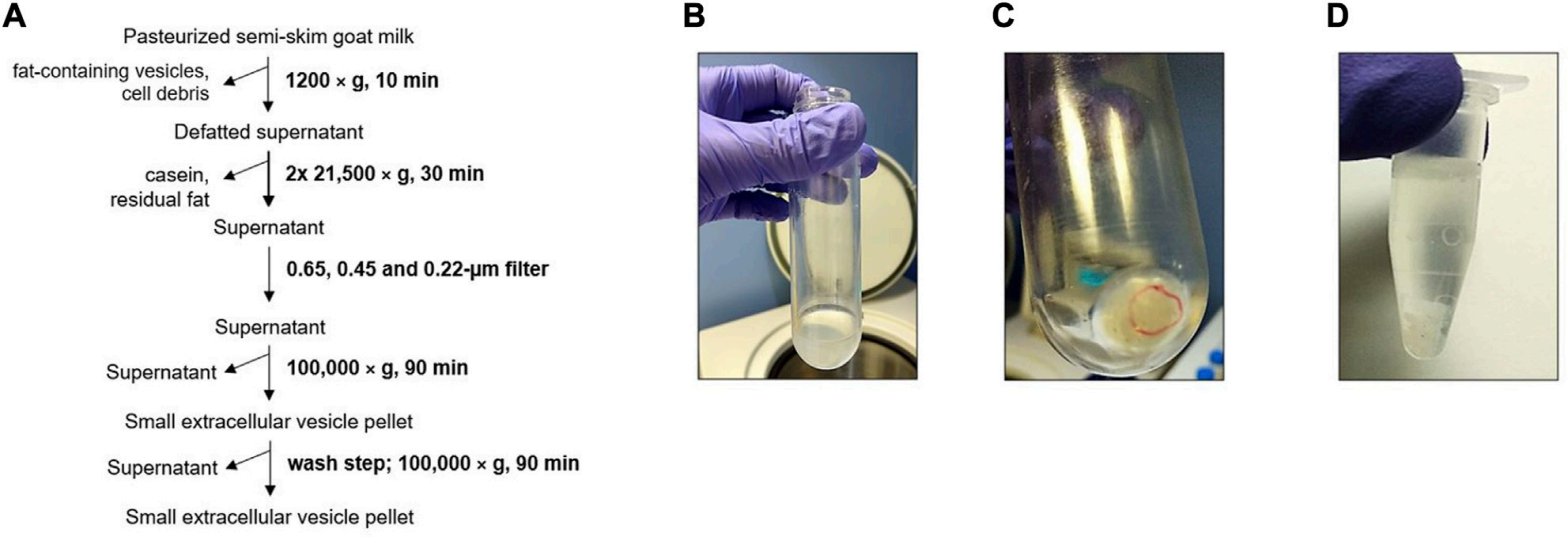
Six-fold differential ultracentrifugation and filtration (physical 6-dUC) procedure. **(A)** Scheme of goat milk small extracellular vesicle (sEV) isolation protocol. **(B)** Appearance of supernatants prior to sEV precipitation. **(C)** Appearance of sEV pellet. **(D)** Milk sEVs suspended in 1X PBS.

Finally, the isolation of sEVs with the physical 6-dUC procedure (Figure 4A) produced a more transparent milk whey after centrifugations and filtrations than did the physical 3-dUC procedure, although the whey was cloudier than following the biophysical procedure (Figure 4B). Nevertheless, the sEV pellets were analogous to snowflakes and adhered strongly to the centrifuge tubes, hindering their resuspension in 1X PBS (Figures 4C, D).

### 3.2 Physicochemical characterization of milk sEVs

The biophysical procedure rendered 3.77 ± 0.81 mg/mL of total protein, quantified by colorimetric assay. The physical 3-dUC and physical 6-dUC protocols yielded 19.67 ± 1.29 mg/mL and 10.12 ± 0.53 mg/mL, respectively.

**FIGURE 5:**
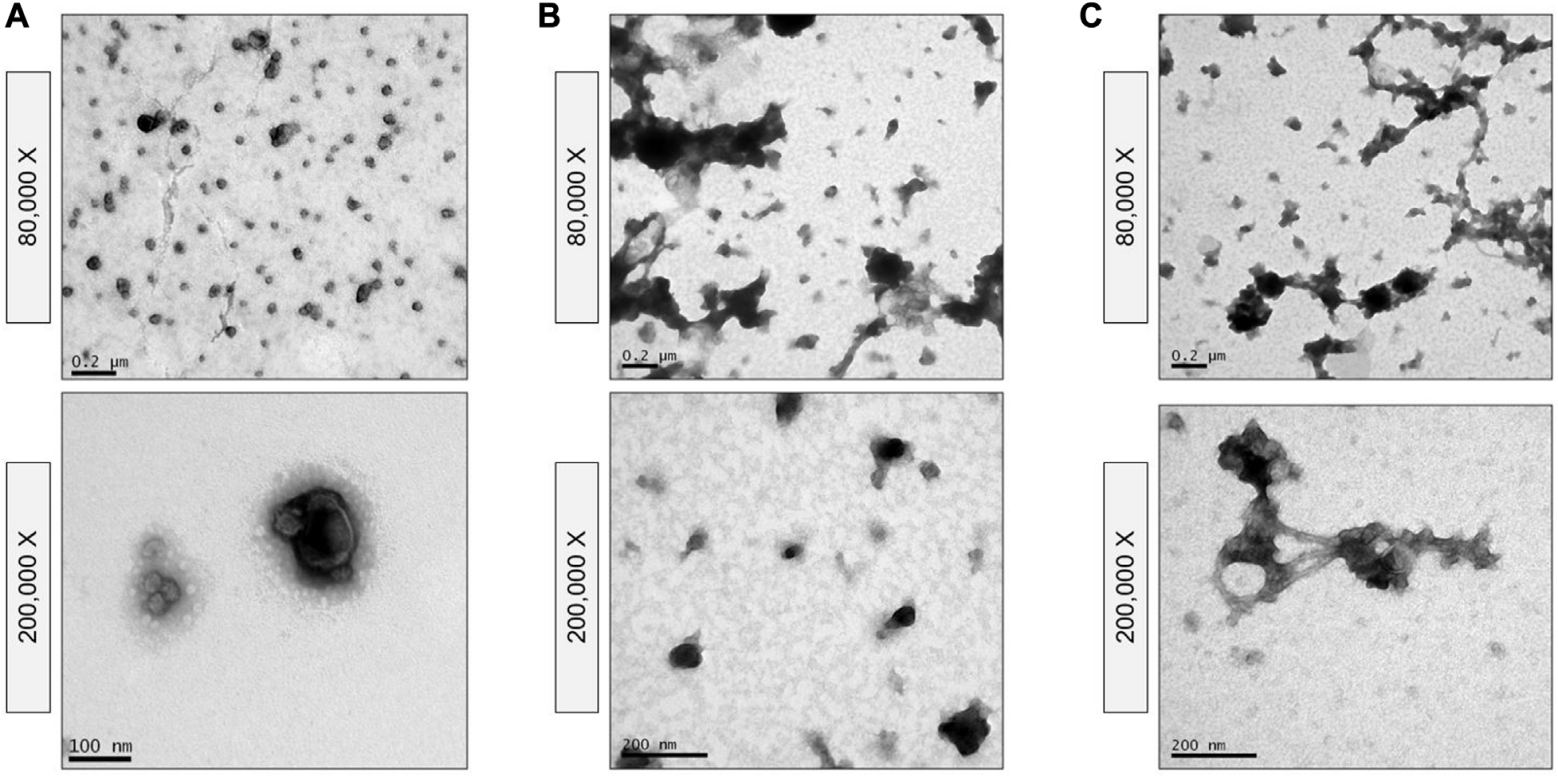
Transmission Electron Microscopy of vesicles isolated by **(A)** the biophysical procedure, **(B)** the physical 3-dUC protocol, and **(C)** the physical 6-dUC procedure. The images show the isolated vesicle population at different levels of magnifications and confirm the presence of protein contaminants and the high state of aggregation of samples obtained through the physical 3-dUC or physical 6-dUC procedure.

TEM confirmed the morphological and size features of the sEVs as well as the homogeneity of the population of isolated vesicles. Nanovesicles isolated by the biophysical procedure presented sEV-like characteristics, such as a lipid bilayer structure and cup-shaped appearance (Figure 5A). This protocol provided a highly concentrated and homogeneous suspension, without aggregates or protein clusters (Figure 2B and Figure 5A), although a small amount of milk lipoproteins [low density, <60 nm (Brennan et al., 2020; Sedykh et al., 2020)] was detected. In contrast, heterogeneous populations of vesicles were isolated following the physical 3-dUC (Figure 5B) and physical 6-dUC procedures and contained large protein aggregates (Figure 5C). The cup-shaped morphology could not be clearly identified in these samples at higher magnifications due to the aggregation of protein residues on the vesicles (Figures 5C, D).

**FIGURE 6:**
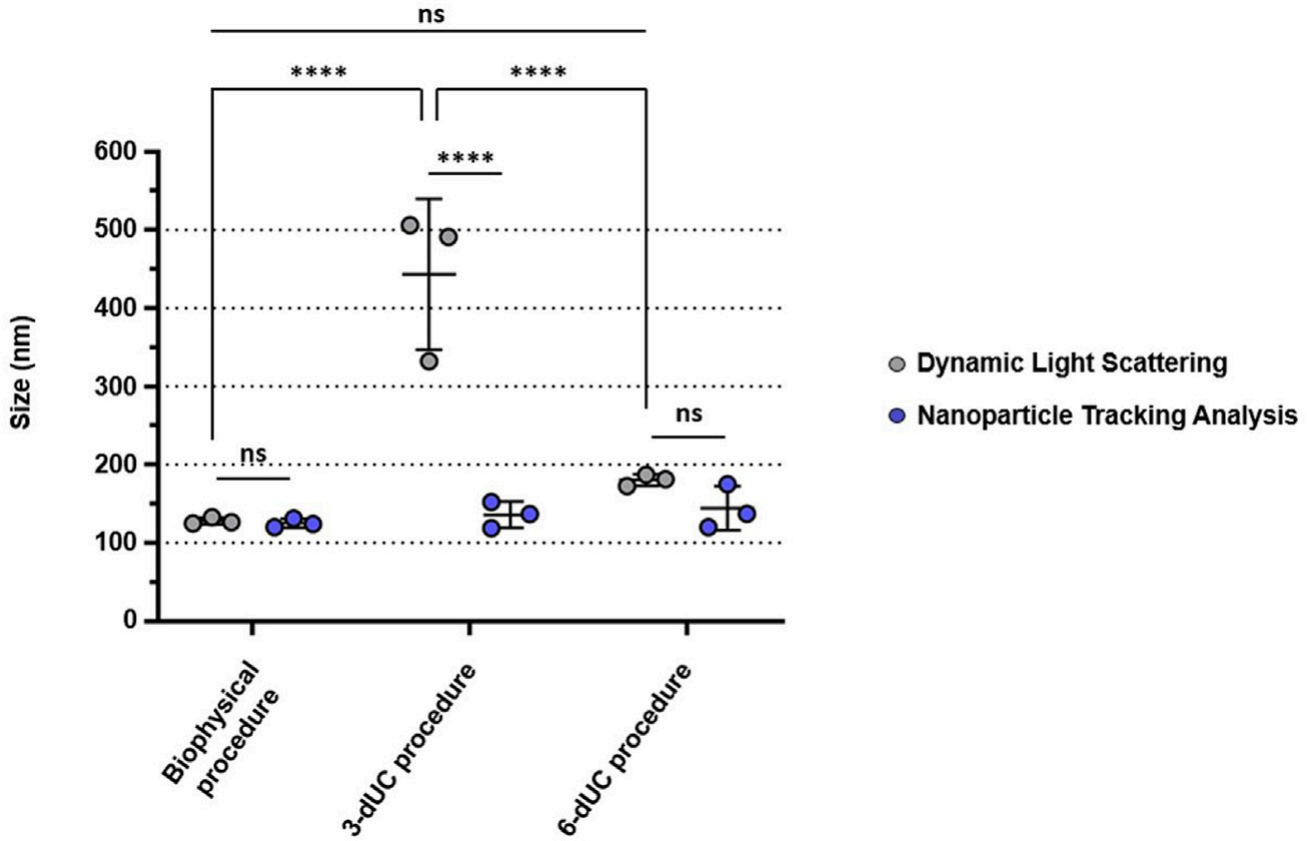
Size comparisons of the isolated small extracellular vesicles, measured by Dynamic Light Scattering (gray) or Nanoparticle Tracking Analysis (blue). The vesicles isolated by the biophysical procedure are the closest in size to sEVs, in comparison with those isolated by the exclusively physical isolation protocols. Data are expressed as the mean ± standard deviation, and the threshold for significance was set at *p* < 0.05.

The size profiles of the nanovesicles was determined by DLS and NTA. sEVs isolated by the biophysical procedure displayed a hydrodynamic size of 128.14 ± 4.13 nm, matching the result achieved by NTA after 0.45-µm syringe filtration (125.30 ± 5.60 nm) (Figure 6 and Supplementary Figure S1). In contrast, nanoparticles isolated with the physical 3-dUC protocol were significantly larger, with hydrodynamic size of 443.18 ± 96.35 nm (two-way ANOVA, *p* < 0.05). Although significant differences were not statistically measured between the biophysical procedure vs the physical 6-dUC protocol, nanoparticles isolated by the latter also showed a larger hydrodynamic size (180.46 ± 7.38 nm). The size distribution showed a reduction after filtration, presenting a modal size of 136.10 ± 16.87 nm for the physical 3-dUC procedure samples and 144.23 ± 28.08 nm for the physical 6-dUC procedure, as measured by NTA (Figure 6 and Supplementary Figures S2–S4).

The polydispersity index (PdI) was also different for the three samples: vesicles from the biophysical procedure presented lower values (PdI: 0.08 ± 0.02) compared with the physical 3-dUC (PdI: 0.36 ± 0.02) and physical 6-dUC (PdI: 0.22 ± 0.01) procedures.

**FIGURE 7:**
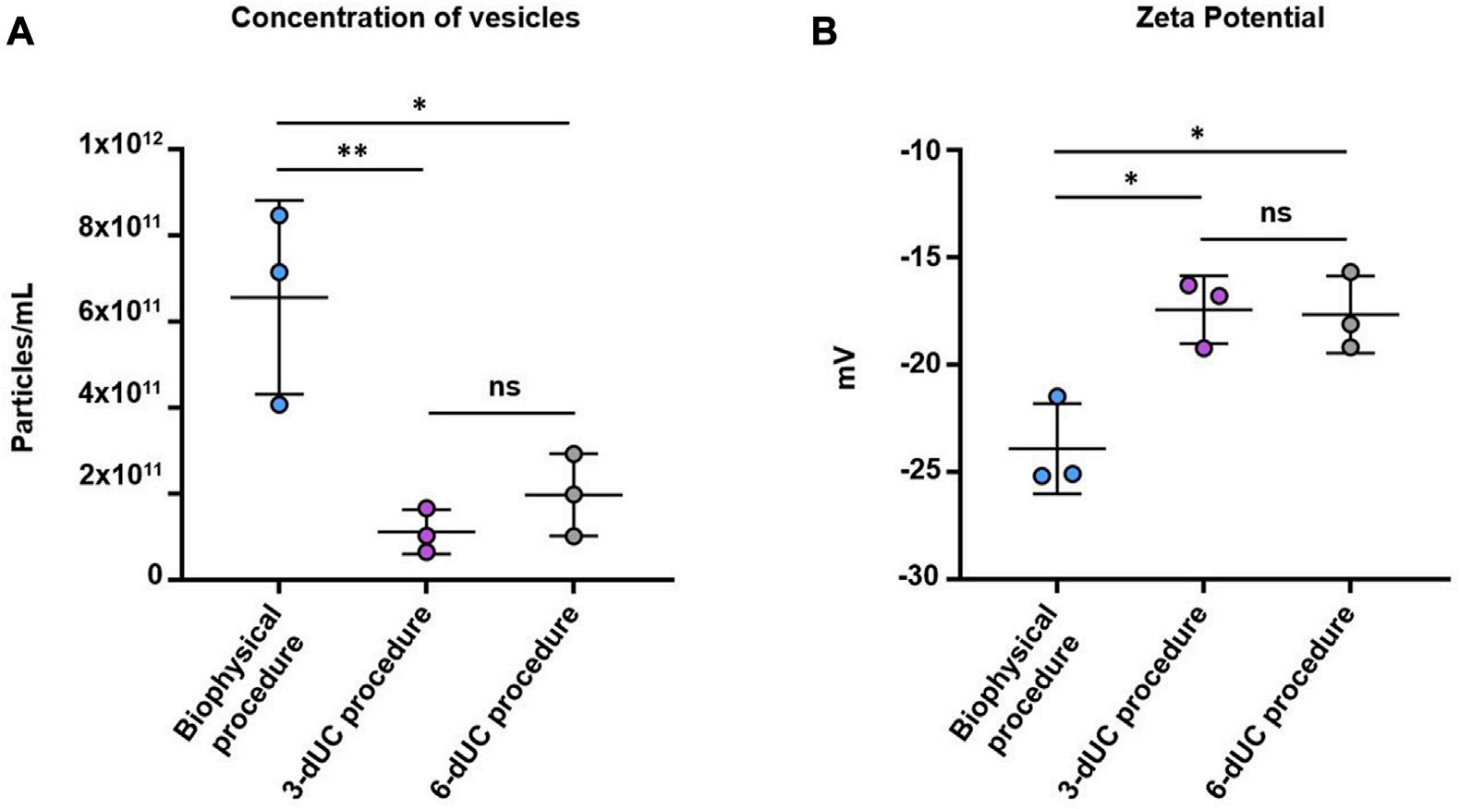
Quantification of **(A)** particle concentration by Nanoparticle Tracking Analysis and **(B)** measurement of the superficial charge by Z-Potential. Data are expressed as the mean ± standard deviation, and the threshold for significance was set at *p* < 0.05.

NTA confirmed the higher sEV content of the sample isolated by the biophysical procedure (6.56 ± 2.25 × 10^11^ particles/mL). Lower values were recorded in the samples obtained by the physical 3-dUC (1.11 ± 0.57 × 10^11^ particles/mL) and physical 6-dUC (1.97 ± 0.96 × 10^11^ particles/mL) procedures (Figure 7A), showing statistical significance (one-way ANOVA, *p* < 0.05).Finally, the colloidal stability of the samples was assessed by measuring the Z-potential. All sEV suspensions were negatively charged (Figure 7B). Measurements from samples isolated by the physical 3-dUC (−17.45 ± 1.58 mV) and physical 6-dUC (−17.68 ± 1.79 mV) procedures were significantly lower (one-way ANOVA, *p* < 0.05) than those from the biophysical procedure (−23.93 ± 2.10 mV).

### 3.3 Western blot analysis

**FIGURE 8:**
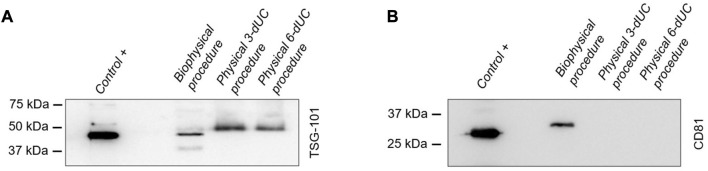
Identification of small extracellular vesicle protein markers by Western blot analysis. The detection of biomarkers **(A)** TSG101 and **(B)** CD81 in milk vesicles was compared with that in sEVs derived from melanoma cells used as a positive control.

In Western Blots, the products of all three milk sEV isolation methodologies displayed a band at 44 kDa for the TSG101 biomarker (Figure 8A). However, in the evaluation of the CD81 biomarker, a band at 26 kDa appeared only in sEVs isolated by the biophysical procedure (Figure 8B).

## 4 Discussion

In this work, we present an optimized methodology for the isolation of sEVs from commercial goat milk by combining the gold standard physical techniques of dUC and SEC with the biological treatment of milk using microbial rennet to obtain highly pure and enriched sEV suspensions.

Several already established dUC protocols use a final ultracentrifugation at 4°C and ≥100,000 × g to precipitate sEVs (Munagala et al., 2016; Vaswani et al., 2017; Yamauchi et al., 2019) but differ slightly in the times and speeds applied in prior centrifugation steps. As there is no single standardized protocol for the collection of exosomes from milk, and considering the high fat and casein content of goat milk (Izumi et al., 2013), we included a number of centrifugation steps to remove as many milk “contaminants” (fat-containing vesicles, cell debris, and large EVs) as possible before precipitating the sEVs.

A previous work (Rahman et al., 2019) proposed to eliminate other non-sEV components during dUC, such as casein and small milk proteins, by acidification with chemical reagents. Although this approach significantly reduces the residual protein content, it also affects the surface composition of the nanovesicles (Rahman et al., 2019). For this reason, we explored the treatment of goat milk with microbial rennet, a biological agent that triggers the coagulation of casein at the natural milk pH. First, the enzymatic activity of rennet hydrolyses casein, which is re-structured into insoluble micelles. Ca^2+^ ions present in the rennet formulation cause the micelles to aggregate and form a gel-like structure, which can be precipitated by centrifugation (Abada, 2019; University of Guelph Enzymic Coagulation of Milk, 2021). The biological treatment with rennet seems essential in the case of goat milk, due to its high casein content (Izumi et al., 2013) and the similar colloidal characteristics of casein micelles and EVs (Somiya et al., 2018). The combination of this biological approach with dUC combined with SEC lessens the aggregation of isolated nanovesicles and preserves their inherent biophysical properties (Sidhom et al., 2020; Yang et al., 2020).

After the complete physicochemical characterization of isolated nanovesicles, we concluded that particles with sEV-like features can be successfully collected by following our optimized approach. TEM images showed vesicles of the size and cup-shaped morphology typical of negatively stained sEVs (Théry et al., 2009). The visualized nanovesicles were not aggregated, and the presence of residual protein or clusters was ruled out. The polydispersity index and size range of the nanovesicles, as established by DLS and NTA, also confirmed the homogeneity of the population and the lack of aggregation.

Western blot analysis confirmed the nature of the isolated nanovesicles, and protein quantification proved the protein enrichment of the suspension as well as the reproducibility of the methodology. These two conclusions were also supported by NTA through the detection of high amounts of vesicles in all measurements.

To assess how our methodology improved the isolation of goat milk sEVs, we compared it with the published physical 3-dUC and physical 6-dUC procedures designed for the isolation of EVs from human breast and bovine milk, respectively. Apart from introducing rennet treatment, our protocol differs from the others mainly by its use of membrane filters with specific molecular weight cut-offs to remove contaminants larger than nanovesicles. Although this methodology reduces the need for centrifugation steps to remove non-sEV elements, it may not be as effective in removing flexible contaminants, which can pass through filters with even smaller pore sizes (Li et al., 2017).

Notable qualitative differences in appearance were observed in the nanovesicles collected by the three methodologies. The pellets of isolated particles from the physical 3-dUC and physical 6-dUC procedures looked aggregated at first glance, probably due to the copious coprecipitation of fat components and milk lipoproteins. The difficulty in resuspending these pellets could affect the exclusive collection of small extracellular vesicles, resulting in carryover of contaminants and other co-isolated vesicular populations that could influence the quantitative results obtained after the characterization of the samples. Based on this fact, we could conclude that the protein content was overestimated, as is true of low-purity EV samples (Théry et al., 2018). This conclusion is supported by the NTA results: higher vesicle content was measured in samples from our biophysical procedure, although the amount of protein was lower than it was in samples isolated by the other two methodologies. This means that a large amount of residual protein was co-isolated and that fewer pure vesicles were collected following the physical 3-dUC and physical 6-dUC protocols. TEM images also supported these conclusions by exhibiting a high level of aggregation with the physical 3-dUC and physical 6-dUC procedures, probably caused by the binding of co-isolated proteins to the surface of sEVs.

The adhesion of residual milk proteins to the surface of sEVs also affected their electronegativity, as assessed by Z-potential, with lower values for the physical 3-dUC and physical 6-dUC procedures. This finding may be explained by changes in the surface composition and could influence the stability and biological activity of isolated particles (Midekessa et al., 2020). Regarding the particle size of the vesicles isolated following the physical 3-dUC and physical 6-dUC procedures, comparison of the DLS with the NTA results indicates that aggregation was reduced after filtering the samples for NTA analysis, which resulted in a decrease in average size. However, the size profile of these vesicles was still larger than that recorded for sEVs isolated by the biophysical protocol, even after filtration.

Finally, the presence of TSG101 and CD81 biomarkers in the samples isolated by the three methods was evaluated to determine the nature of the vesicles. TSG101 was detected in all of them but CD81 was identified only in the vesicles isolated by the biophysical protocol. Previous studies have shown that TSG101 is commonly found in different vesicle types secreted by cells, but sEVs are particularly enriched in CD81 tetraspanins (Andreu and Yáñez-Mó, 2014; Lee et al., 2019). Thus, the lack of CD81 in samples from the physical 3-dUC and physical 6-dUC procedures could indicate that the isolated vesicle population does not specifically correspond to sEVs. This could be confirmed with a more extensive biomarker panel or proteomics studies.

Despite the promising application of our methodology, some limitations could still be addressed in further studies. One of these possible limitations is the co-isolation of small lipoprotein vesicles with the sEVs, as detected by TEM. They are a common contaminant of milk sEV suspensions, due to their similar colloidal characteristics. However, samples collected by use of our combined methodology with microbial rennet were highly enriched in a homogeneous and stable population of goat milk sEVs, with no alterations of expected physicochemical properties. Thus, our protocol overcomes the problems associated with the treatment of milk samples with acid reagents for the elimination of residual milk proteins. On the other hand, a more specific study of lipids and/or proteins could establish whether there is any biochemical influence of the rennet on the enzymes or proteins present in the vesicular membrane. In addition, it would be interesting to evaluate the applicability of this methodology to the isolation of sEVs from other milk sources such as bovine, sheep, or human.

In summary, our methodology enables the isolation and purification of small extracellular vesicles from goat milk, through the combination of traditional physical techniques with simple and inexpensive biological ones. The isolated sEVs present the usual properties catalogued for these vesicles, from a physicochemical point of view and in relation to some of their most characteristic surface biomarkers. The high isolation yields as well as the high grade of purity emphasize the usefulness of goat milk as an economic and enriched source of sEVs in comparison with other biological fluids and cell culture media (Théry et al., 2006). In addition, we chose to isolate these sEVs from goat milk using a commercial product due to its accessibility and standardization among grocery stores. Nevertheless, the methodology developed in this study could also be directly applied to milk sera, a waste product of the cheese-producing food industry. This could have a positive environmental impact on the recycling of these milk sera, which are normally discarded, thus improving the environmental sustainability of our goat milk sEVs isolation protocol.

To support the potential use of these sEVs in the diagnostic and therapeutic field, we would like to highlight that several biological assays, including proteomics, *in vitro* cytotoxicity and metabolic activity, biocompatibility and *in vivo* toxicity have been previously reported by our group in already published works (Santos-Coquillat et al., 2022).

## Data Availability

The raw data supporting the conclusion of this article will be made available by the authors, without undue reservation.
