# Supplementary material for: Isolation of goat milk small extracellular vesicles by novel combined bio-physical methodology
Source: Front Bioeng Biotechnol. 2023 Sep 27;11:1197780. doi: 10.3389/fbioe.2023.1197780 (PMC10564981; doi:10.3389/fbioe.2023.1197780)
Supplement: Supplementary file 1 [file DataSheet1.PDF]

Supplementary Material

Isolation of Goat Milk Small Extracellular Vesicles by Novel Combined Bio-physical Methodology

María Isabel González, Begoña Gallardo, Carlos Cerón, Elena Aguilera-Jiménez, Marta Cortes-Canteli, Héctor Peinado, Manuel Desco*, Beatriz Salinas*

***Correspondence:** Manuel Desco and Beatriz Salinas; desco@hggm.es, bsalinas@hggm.es

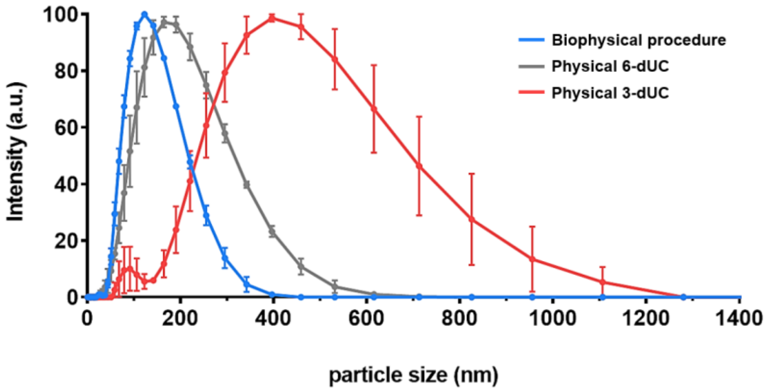

Supplementary Figure 1. Representative Dynamic Light Scattering (DLS; n = 3) of vesicles isolated by Biophysical procedure (blue), Triple differential centrifugation and filtration procedure (physical 3-dUC; red) and Six-fold differential ultracentrifugation and filtration procedure (physical 6-dUC; grey).

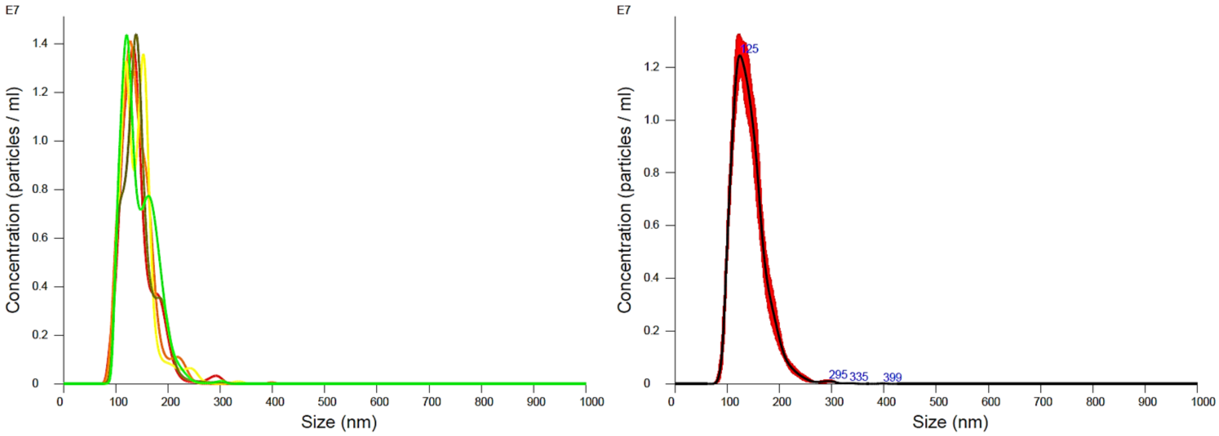

Supplementary Figure 2. Representative Nanoparticle Tracking Analysis (NTA) of vesicles isolated by Biophysical procedure. Finite track length adjustment (FTLA) concentration/size graph (left, n = 5 records) and averaged FTLA Concentration/size graph (right).

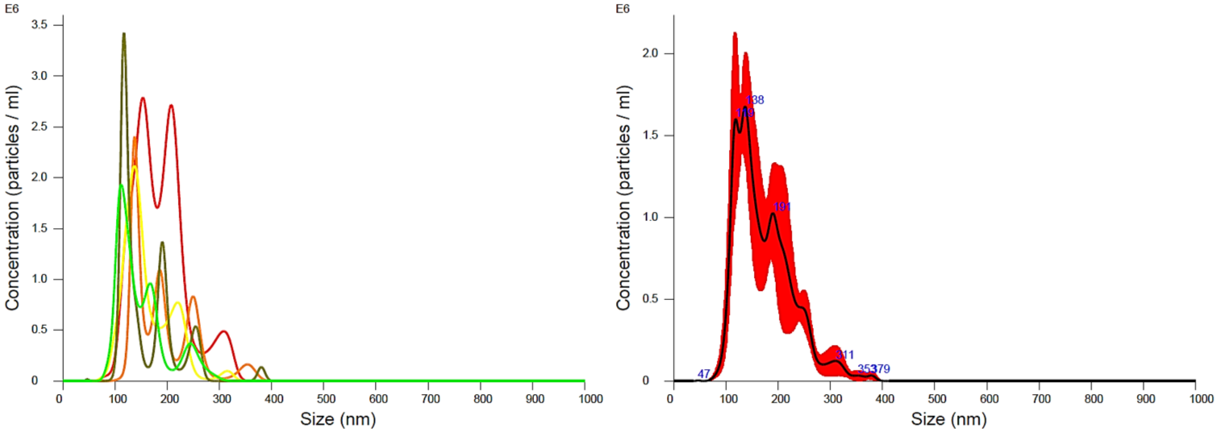

Supplementary Figure 3. Representative Nanoparticle Tracking Analysis (NTA) of vesicles isolated by Triple differential centrifugation and filtration (physical 3-dUC) procedure. Finite track length adjustment (FTLA) concentration/size graph (left, n = 5 records) and averaged FTLA Concentration/size graph (right).

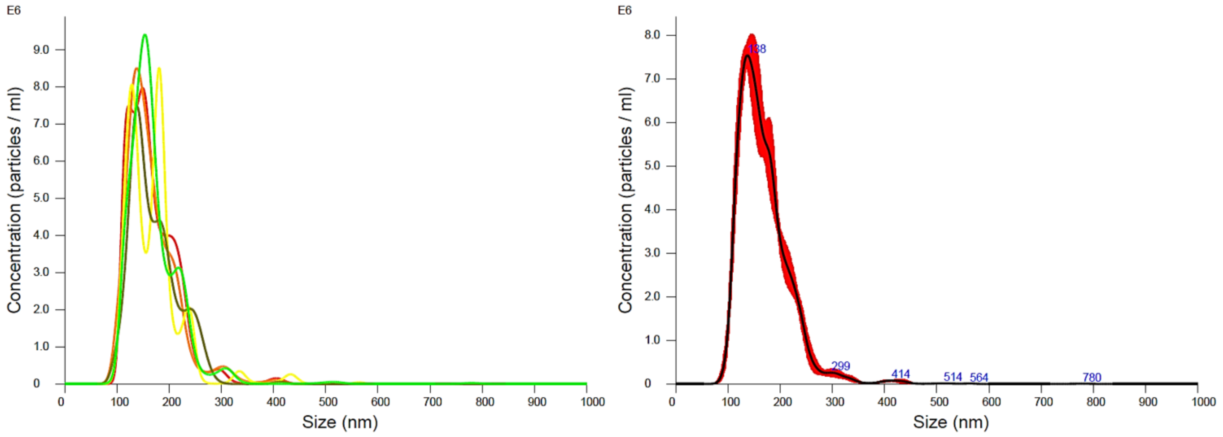

Supplementary Figure 4. Representative Nanoparticle Tracking Analysis (NTA) of vesicles isolated by Six-fold differential ultracentrifugation and filtration (physical 6-dUC) procedure. Finite track length adjustment (FTLA) concentration/size graph (left, n = 5 records) and averaged FTLA Concentration/size graph (right).
